# The effect of sodium bicarbonate mini-tablets in a carbohydrate hydrogel on prolonged high-intensity cycling performance and metabolism in acute normobaric hypoxia

**DOI:** 10.1007/s00421-025-06069-6

**Published:** 2026-01-03

**Authors:** Eli Spencer Shannon, S. Andy Sparks, Lars Robert McNaughton, Kelly Marrin, Craig Alan Bridge

**Affiliations:** 1 The Sport and Exercise Performance Enhancement and (P)rehabilitation (S- PEP) Group, Edge Hill University, Ormskirk, UK; 2 Maurten AB, Gothenburg, Sweden; 3 Research Institute for Sport and Exercise Sciences, Liverpool John Moores University, Liverpool, UK; 4 Consultant in Sport, Nutrition, and Exercise Science, Southport, UK PR8 6JQ

**Keywords:** Alkalosis, Altitude, Extracellular buffer, Endurance, Fatigue

## Abstract

**Purpose:**

Hydrogen cation (H^+^) accumulation is exacerbated in hypoxia. Sodium bicarbonate (NaHCO_3_) ingestion can reduce fatigue associated with high-intensity exercise performance by counteracting H^+^ ion accumulation. The present study aimed to determine the effect of 0.3 g·kg^− 1^ NaHCO_3_ mini-tablets in a carbohydrate hydrogel on 40 km cycling time trial (TT) performance in trained male cyclists in acute hypoxia.

**Methods:**

Fourteen trained male cyclists completed a ramp test to determine VO_2Peak_. After, in a randomised, double-blinded, placebo-controlled crossover study, participants completed three 40 km cycling TTs (1 x familiarisation, 2 x experimental) in acute hypoxia (16.7 ± 0.2 FiO_2_). Each experimental TT followed 90 min after the ingestion of 0.3 g·kg^− 1^ BM NaHCO_3_ or an appearance-matched placebo.

**Results:**

After NaHCO_3_ ingestion, 40 km cycling TT performance was improved by 1.2% in comparison to placebo (*t =* 3.84, *p* = 0.02, *g* = 0.19). Additionally, NaHCO_3_ ingestion raised blood pH (*f* = 48.47, *p* = < 0.001, pη^2^ = 0.79) and blood HCO_3_^−^ (*f* = 64.13, *p* = < 0.001, pη^2^ = 0.84) pre-exercise and throughout the TT. Aggregated gastrointestinal symptoms (GIS) were minimal, and did not differ between conditions (NaHCO_3_, 110 AU; Placebo, 119 AU; *z* = 0.38, *p* = 0.71, *r* = 0.10).

**Conclusion:**

The present study suggests that 0.3 g·kg^− 1^ NaHCO_3_ can enhance 40 km cycling TT performance in acute hypoxia, likely a result of improved buffering capacity. Cyclists competing at altitude could consider the ingestion of this form of NaHCO_3_ to minimise the deleterious effects of acute hypoxia.

## Introduction

Hypoxic environments are commonly utilised by athletes to enhance training adaptations and performance. Both natural and simulated exposure to hypoxic environments can induce beneficial physiological responses; however, acute hypoxic exposure impairs performance due to the reduced barometric pressure, which decreases the alveolar partial pressure of oxygen (PO_2_) (Koehle et al. 2014). Consequently, the O_2_ delivery to the active skeletal musculature is hindered which increases the reliance on non-oxidative metabolism (Bassett and Howley 2000) and produces an ergolytic effect, resulting in a ~ 6.5% reduction in performance per 1000 m in elevation for time trial (TT) efforts longer than 10 min (Deb et al. 2018a). To attenuate the ergolytic effects of acute hypoxia during important competition, athletes often attend training camps that incorporate altitude acclimatisation strategies, such as “live high train low” (LHTL) to enhance performance at both altitude and sea level by stimulating erythropoietin (EPO) production and increasing haemoglobin mass (HB_mass_) (Gore et al. 2013). However, popular training camp destinations such as Colorado Springs, USA (1839 m), Kühtai, Austria (2017 m) and Livigno, Italy (1816 m) are generally not conducive to a true LHTL approach due to the lack of nearby locations below 1200 m in elevation that would allow training at a “low” elevation. It is therefore important to establish if alternative or complimentary acute ergogenic strategies can be effective in these environments to improve performance.

Although debated (Westerblad 2016), the accumulation of H^+^ ions leading to a reduced intracellular pH is generally associated as the main determinant of skeletal muscle fatigue in high intensity exercise, which is exacerbated in acute hypoxia (Adams and Welch 1980). One logical strategy to mitigate the acidosis response in acute hypoxia is the ingestion of extracellular buffers. Consuming extracellular buffering agents induces blood alkalosis by increasing blood HCO_3_^−^ and blood pH, which enhances intramuscular H^+^ efflux (Requena et al. 2005), thereby delaying fatigue during exercise that predominantly relies on glycolytic metabolism (Siegler et al. 2016). A collection of extracellular buffering agents has therefore been researched to determine their efficacy on high-intensity exercise performance such as sodium phosphate (Folland et al. 2008), sodium citrate (Cerullo et al. 2020) and sodium bicarbonate (NaHCO_3_) (Grgic et al. 2021). However, NaHCO_3_ is the most commercially available extracellular buffer and is widely regarded as the most effective extracellular buffer (de Oliviera et al. 2021). As a result, this alkalising agent has demonstrated its ergogenicity in short high-intensity exercise up to 10 min in duration including 2 km rowing performance in female CrossFit^®^ athletes (Martin et al. 2023) and 4 km cycling TT performance in trained male cyclists (Gough et al. 2018b; Hilton et al. 2020a). Despite this, its ergogenic effects on prolonged high-intensity exercise performance are less clear.

The ingestion of NaHCO_3_ has demonstrated its ergogenicity in prolonged high-intensity exercise performance exceeding 10 min in duration (McNaughton et al. 1999; Egger et al. 2014); however, this is not always observed (Stephens et al. 2002; Freis et al. 2017). The equivocal results reported in these studies are likely due to contrasting study designs inclusive of differing exercise protocols, timing of ingestion, and the form by which NaHCO_3_ is administered. As such, NaHCO_3_ dissolved in aqueous solution and encapsulated with a gelatin/vegetarian coating have been found to increase the frequency of gastrointestinal symptoms (GIS) in comparison to alternative ingestion forms (Hilton et al. 2020b; Gough and Sparks 2024a). Strategies to reduce the GIS responses, have been shown to be effective at improving longer duration exercise performance than is generally associated with the ergogenic potential of NaHCO_3_ (Leach et al. 2023). Consequently, the development of commercially available products specifically designed to cause alkalosis whilst limiting the GIS responses have recently become popular. One such product uses NaHCO_3_ mini-tablets ingested in a carbohydrate (CHO) hydrogel (Maurten Bicarb System, Maurten AB, Gothenburg, Sweden), and this has been shown to increase blood HCO_3_^−^, prolong alkalosis (Gough and Sparks 2024a) and improve exercise performance whilst potentially reducing the negative influence of GIS (Gough and Sparks 2024b). As such, the CHO hydrogel acts as a vehicle to deliver the NaHCO_3_ mini-tablets through the pyloric sphincter, reducing these mini-tablets’ interaction with stomach acid whilst minimising the potential for GIS (Gough and Sparks 2024a). Furthermore, Shannon et al. (2024) were the first to observe improvements in 40 km cycling TT performance using this ingestion strategy in trained cyclists at sea level; however, research has yet to examine the effect of this extracellular buffer on prolonged high-intensity exercise performance in simulated altitude.

Whilst the ergogenic effects of NaHCO_3_ ingestion at sea level are well-established, its benefits in hypoxia are less understood. Few studies have demonstrated performance enhancing effects of NaHCO_3_ ingestion in this environment (Deb et al. 2017, 2018b; Gough et al. 2018a, 2019) whereas others have found no benefit (Flinn et al. 2014; Saunders et al. 2014a), and there has been no concerted attempt to examine NaHCO_3_ ingestion on high-intensity cycling TT performance exceeding 4 km in length. Interestingly, except for Saunders et al. (2014a), each of the aforementioned studies has used a simulated altitude of ~ 3000 m, which is an altitude that is unlikely to be experienced by cyclists during TTs or races for prolonged periods, given that most training camps and competition venues are situated at elevations of 1500–2500 m (Carr et al. 2023; Willmott et al. 2024). Research is therefore needed to ascertain the effect of NaHCO_3_ ingestion on ecologically valid elevations using this new ingestion strategy. The aim of the present study was to therefore determine the effect of ingesting NaHCO_3_ mini-tablets in a CHO hydrogel on 40 km cycling TT performance in acute normobaric hypoxia (~ 1850 m) in trained cyclists.

## Methods

### Participants

Fourteen trained male cyclists (age 43 ± 9 years; height 1.78 ± 0.08 m; body mass, 74 ± 7 kg; body fat, 14.8 ± 3.8%; VO_2Peak_, 55.1 ± 5.0 mL·kg^− 1^·min^− 1^; HR_Max_, 177 ± 9 b·min^− 1^; W_Peak_, 378 ± 46 W) completed a medical questionnaire and provided verbal and written informed consent prior to participation in the current study. Ethical approval was attained from the institutional ethics committee and all procedures were carried out in accordance with the Declaration of Helsinki (2013) and its later amendments. A priori power analysis was performed using G*Power (v3 1.9.6, Kiel University, Dusseldorf, Germany). A sample size of twelve was determined to be sufficient to detect a small effect size (0.20), assuming 0.05 error probability and 0.80 power. Participants were defined as apparently healthy and trained > 3 d·week^− 1^, accumulated > 180 km·week^− 1^, and had more than two years cycling experience which led to regularly competing in cycling TT races, specifically in the 40 km (~ 25 mile) TT category. Based on these attributes, participants were defined as ‘trained’ (McKay et al. 2021). Individuals ingesting intra- or extracellular buffering supplements (i.e. beta-alanine, NaHCO_3_) (Gurton et al. 2023), travelled to moderate (2000–3000 m) or high (3000–5500 m) altitude in the past six months (Bärtsch et al. 2008), or were currently prescribed and taking medication were ineligible for participation in this study. Individuals with diagnosed GIS-related disorders, hypertension, or renal impairment were also excluded from participating.

### Experimental study design

**Fig. 1:**
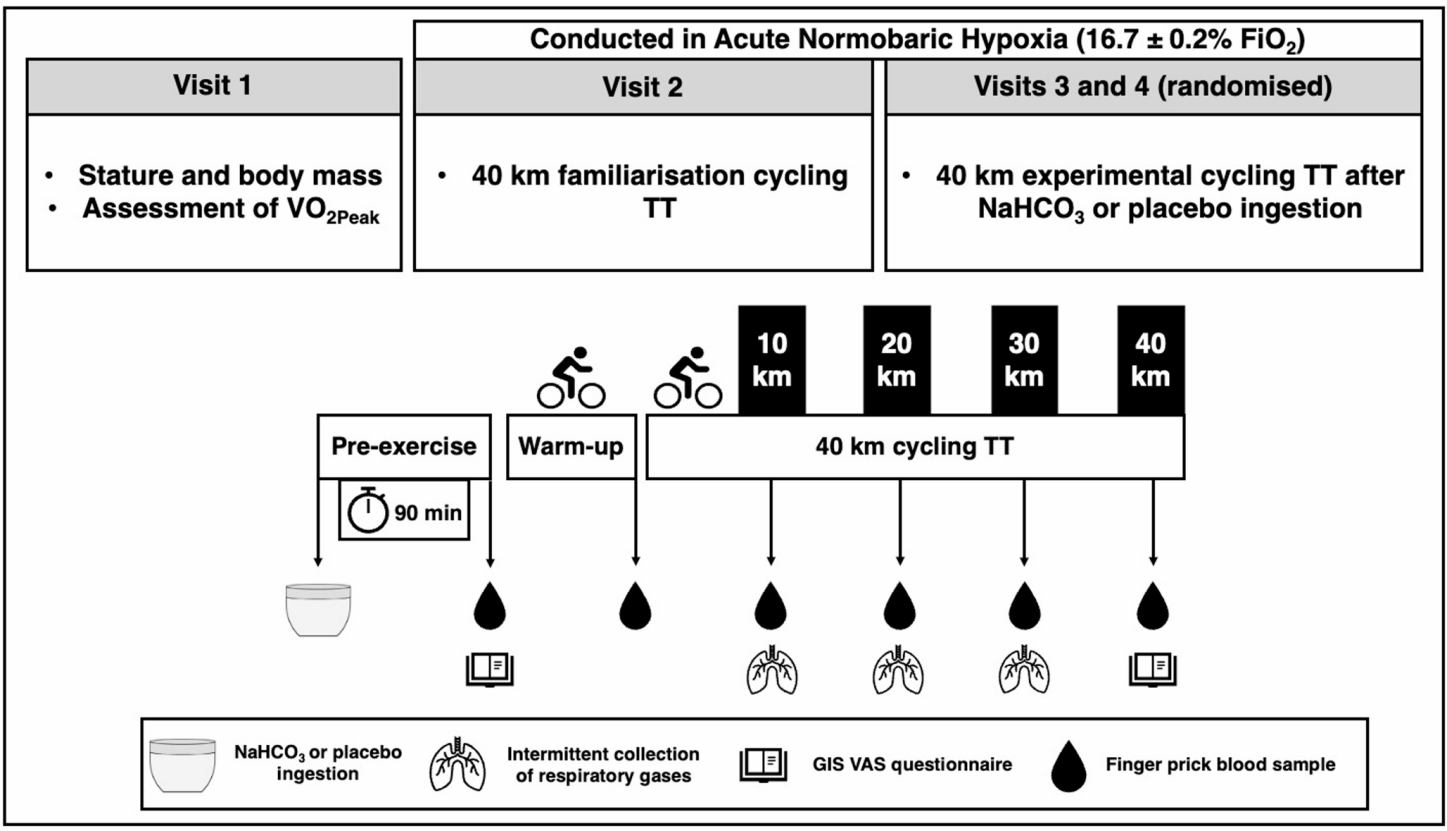
Experimental design and overview of the experimental trials. *VO*_*2Peak*_, peak oxygen uptake; *TT*, time trial; *NaHCO*_*3*_, sodium bicarbonate; *GIS*, gastrointestinal symptoms; *VAS*, visual analogue scale

In a randomised, double-blind, crossover study (Fig. 1), participants attended the laboratory on four separate occasions at the same time of day (± 1 h) (Reilly 1990) separated by two to seven days. During the initial visit, participants completed a general health screening form, measured body composition (body mass and body fat) (BOD POD ^®^, Cosmed, Italy), and completed a maximal graded exercise ramp test to determine peak oxygen uptake (VO_2Peak_) at sea level (20.93% FiO_2_). The second visit required participants to complete a 40 km familiarisation cycling TT on an air-braked cycle ergometer (Wattbike Pro, Nottingham, UK) in a normobaric hypoxic environmental chamber (Model S016r-7-sp, TISS, Portsmouth, UK), which was used to recreate ambient hypoxic conditions with a fractional inspired O_2_ (FiO_2_) of 16.7 ± 0.2% (~ 1850 m above sea level) whilst also accounting for temperature (20 ± 1 °C) and relative humidity (40 ± 5%). These environmental conditions were thereby replicated for the third and fourth visit, which consisted of two experimental 40 km cycling TTs. These TTs proceeded after the ingestion of 0.3 g·kg^− 1^ body mass (BM) NaHCO_3_ or an appearance-matched, maltodextrin placebo. Participants were advised to avoid the consumption of alcohol and taking part in strenuous exercise in the 24 h preceding each visit and were encouraged to arrive to the laboratory well-hydrated. Prior to each visit, participants consumed their usual pre-TT meal (consumed 3.5 h before each TT) and were required to replicate this prior to each trial. As a method to ensure compliance, participants completed a 24 h dietary recall.

### Experimental procedures

#### The determination of peak oxygen uptake (VO_2Peak_)

A maximal graded exercise test was conducted on an electromagnetic cycle ergometer (Lode Excalibur Sport, Groningen, the Netherlands) to determine peak oxygen uptake (VO_2Peak_). Following a 5 min warm-up of cycling at 150 W, participants completed a ramp test by which each participant started cycling at 60 W, followed by an increase of 1 W every 2 s (equivalent to 30 W·min^− 1^) whilst maintaining a preferred cadence of 75–90 rev·min^− 1^ until volitional exhaustion (Deb et al. 2018b). Breath-by-breath pulmonary gases (oxygen uptake, VO_2_; carbon dioxide, VCO_2_; minute ventilation, VE; respiratory exchange ratio, RER) were measured on a clinical metabolic cart (Vyntus, Vyaire Medical, Chicago Illinois, USA). Peak power output (W_Peak_) was defined as the greatest power output achieved at the end of the test, whilst VO_2Peak_ was decided as the highest 30 s rolling average of VO_2_. This was further confirmed when rating of perceived exertion (RPE) (Borg 1982) exceeded 18 arbitrary units (AU) and when RER > 1.15 (Midgley et al. 2007).

#### Ingestion strategy

Upon arrival to the physiology laboratory, participants either ingested 0.3 g·kg^− 1^ NaHCO_3_ mini-tablets (3 mm in diameter, 1.5 mm in height) (Maurten AB, Gothenburg, Sweden) or a taste and appearance-matched placebo consisting of maltodextrin mini-tablets in a CHO hydrogel (~ 40 g). To appearance match both conditions, the placebo mini-tablet dose was equivalent to the participant’s dose of NaHCO_3_ (g) multiplied by 0.825. The 40 g of CHO was combined with 200 mL water, and once mixed, the mini-tablets were added, as per the manufacturer guidelines. Both treatments were ingested 90 min prior to the start of the experimental 40 km cycling TTs, which was based on recent research elucidating that this ingestion time point would induce a sufficient blood HCO_3_^−^ response at pre-exercise (Gough and Sparks 2024a; Shannon et al. 2024). Following the end of each experimental TT, participants were asked to complete a supplement belief questionnaire requesting which supplement was perceived to be ingested (0, no confidence; 5, not sure; 10, highest confidence) whereby any value above 5 was considered a successful detection as per previous research (Gough and Sparks [Bibr CR24][Bibr CR25]).

#### Gastrointestinal symptoms

Participants responded to an adapted visual analogue scale (VAS) (9-item, 10-point Likert scale) to quantify GIS with 0 representing “no symptoms” and 10 being “most severe symptoms” which were reported in arbitrary units (AU) (Carr et al. 2011). These symptoms included stomach bloating, stomach cramp, stomach-ache, belching, bowel urgency, diarrhoea, nausea, flatulence, and vomiting. Total GIS was defined as the sum of symptom scores across each of the 9 aforementioned symptoms for each timepoint (pre-exercise, post-exercise) per condition (NaHCO_3_, placebo). Additionally, aggregated GIS was defined as the sum of symptoms amalgamated between both measured timepoints (total symptoms at pre-exercise + total symptoms at post-exercise), whereas peak GIS, measured by peak severity score, was considered as the greatest symptom rating (0–10, AU) at each timepoint.

#### 40 km cycling time trial (TT) procedures

After arrival at the laboratory, participants rested for 10 min in the environmental chamber to equilibrate the atmospheric and body O_2_ stores (Andreassen and Rees 2005). Blood lactate (5 µL; Lactate 2 Pro, Arkray, Japan), blood HCO_3_^−^, blood pH, electrolytes ([K^+^], [Na^+^], [Ca^2+^]. [Cl^−^]), haemoglobin with oxygen saturation (SpO_2_), and partial pressure of oxygen (PO_2_) and carbon dioxide (PCO_2_) (90 µL; ABL800, Radiometer, Copenhagen, Denmark) were drawn pre-exercise. The data from blood lactate and blood electrolytes were used to calculate the apparent strong ion difference (SID) using the following equation: SID = [K^+^] + [Na^+^] + [Ca^2+^] – [Cl^−^] – [Lac^−^] (Lloyd 2004). Following this, participants selected preferred frame geometry (handlebar and saddle) which was recorded and replicated for the later trials. Each warm-up was individualised (5–10 min) and replicated for each TT. Additional blood samples (5 µL and 95 µL) were drawn from the participant’s forefinger following the completion of each individualised warm-up.

Throughout the 40 km TT, respiratory responses (VE, VO_2_, VCO_2_, and RER) were measured at 9–10 km, 19–20, and 29–30 km using the methods previously described. Blood parameters, heart rate (Forerunner 15, Garmin, Kansas City, US), and RPE were recorded at the end of each quartile of the TT. Participants were instructed to cycle 40 km TT as quickly as possible, with no visual or physiological feedback except for distance covered. During the TT, participants could self-select their pedalling cadence and adjust the air-resistance using a lever on the left side of the cycle ergometer in order to complete the TT as quickly as possible. An electric fan (Clarke Air, Essex, UK) was placed 1.5 m from the cycle ergometer to promote evaporative cooling throughout the duration of the 40 km TT using the same maximal setting for every trial. Overall and quartile performance variables of time to complete, power output, speed, and cadence were recorded. In the current study, “pacing strategy” referred to the total distribution of power output across the duration of the TT (i.e. how participants changed their effort over time).

### Statistical analysis

All data were assessed for normality and homogeneity of variance using the Shapiro-Wilk Test and Mauchly Test, respectively. Overall TT performance (time to complete, overall mean power output, and overall mean speed) were analysed using a paired t-test. Since *n* ≤ 20, effect sizes were calculated using Hedge’s *g* [(Mean_1_ – Mean_2_) / SD_Pooled_)] (Lakens 2013) and were interpreted as trivial (< 0.20), small (0.20–0.49), moderate (0.50–0.79), or large (> 0.80) (Cohen 1988). All GIS data were reported in arbitrary units (AU) (aggregated, peak, and total) and violated the assumption of normality. Therefore, a Wilcoxon signed rank test was performed as the non-parametric replacement to analyse the GIS response data. Effect sizes (*r)* were interpreted as small (0.10), medium (0.24), and large (0.37) and were calculated as r = z/√n (Ivarsson et al. 2013). Performance variables (power, speed, cadence), blood parameters, cardiorespiratory responses, and perceptual responses were detected through a two-way repeated measures analysis of variance (ANOVA) using a Bonferroni correction. These effect sizes (pη^2^) were considered as small (0.01), medium (0.06), or large (0.14) (Cohen 1988). All data was analysed in the Statistical Package for Social Sciences (IBM SPSS, version 29, Chicago, Illinois, US) whereby significance was set at *p* < 0.05, and all data was presented as mean ± SD unless stated otherwise.

## Results

### 40 km cycling time trial performance

**Fig. 2:**
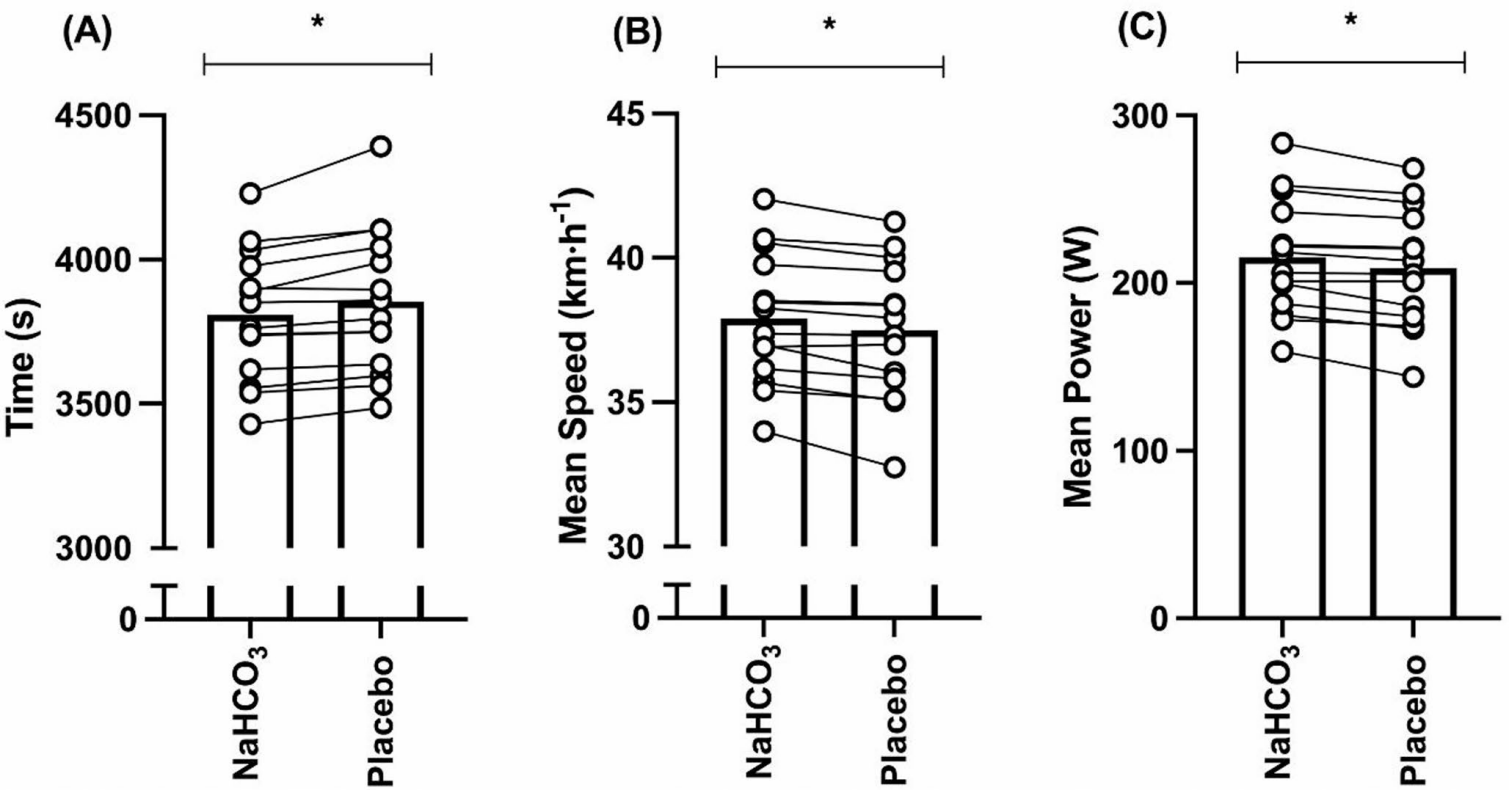
Individual and mean responses for time (A), mean speed (B), and mean power (C) for the time trials following sodium bicarbonate (NaHCO_3_) and placebo ingestion. * denotes significant condition difference (*p* < 0.05)

**Fig. 3:**
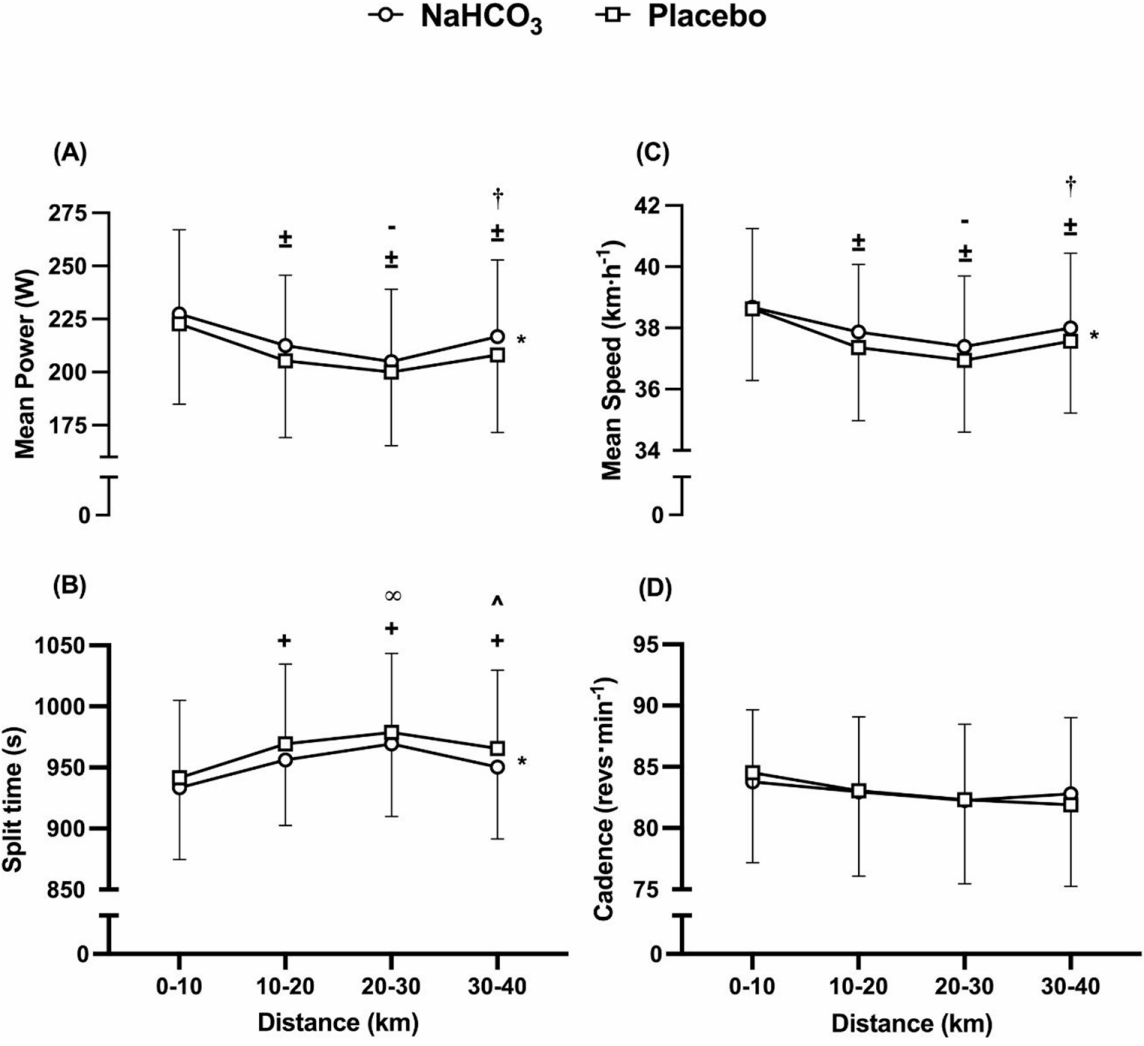
Mean (± SD) mean power (A), split time (B), mean speed (C), and cadence (D) responses to sodium bicarbonate (NaHCO_3_) and placebo ingestion for each quartile of the time trials. **+** denotes significant increase from first timepoint (0–10 km) (*p* < 0.01), **∞** denotes significant increase from second timepoint (10–20 km) (*p* < 0.001), ^ denotes significant decrease from third timepoint (*p* < 0.001) (20–30 km), **±** denotes significant decrease from first timepoint (0–10 km) (*p* < 0.05), **-** denotes significant decrease from second timepoint (10–20 km) (*p* < 0.001), and † denotes significant increase from third timepoint (20–30 km) (*p* < 0.001). * denotes significant difference between conditions (*p* < 0.05)

Overall performance time was enhanced by ~ 45 s (*t =* 3.84, *p* = 0.02, *g* = 0.19) following the ingestion of NaHCO_3_ (3809 ± 226 s) in comparison to placebo (3855 ± 252 s), equating to a 1.2% improvement (Fig. 2a). In addition, the overall mean speed was faster (*t* = 4.19, *p* = 0.001, *g* = 0.18) during the NaHCO_3_ trial (37.91 ± 2.28 km·h^− 1^) compared to placebo (37.49 ± 2.38 km h^− 1^), whilst a ~ 6 W improvement was observed for overall mean power output (*t* = 4.62, *p* = < 0.001, *g* = 0.18) (Fig. 2bc) was observed during the NaHCO_3_ trial (215 ± 35 W) in contrast to placebo (209 ± 36 W). Furthermore, the quartile split time was faster in the NaHCO_3_ trial in comparison to placebo (*f* = 14.77, *p* = 0.02, pη^2^ = 0.53), whilst the quartile mean speed (*f* = 17.56, *p* = 0.001, pη^2^ = 0.58) and quartile mean power (*f =* 21.33, *p* = < 0.001, pη^2^ = 0.62) were both raised following NaHCO_3_ consumption compared to placebo (Figs. 3a-c). Quartile split time (*f* = 16.61, *p* = < 0.001, pη^2^ = 0.82) mean speed (*f* = 21.94, *p* = < 0.001, pη^2^ = 0.63) and mean power output (*f* = 16.98, *p* = < 0.001, pη^2^ = 0.82) changed considerably throughout the TT, with no significant interaction effects observed for either of these variables (mean power, *f* = 0.94, *p* = 0.46, pη^2^ = 0.20; mean speed, *f* = 0.89, *p* = 0.48, pη^2^ = 0.20; split time, *f* = 0.66, *p* = 0.60, pη^2^ = 0.15), suggesting that the pacing strategy was unaffected by the experimental conditions. Despite the improvement in TT performance following NaHCO_3_ ingestion, cadence was unaffected by condition (*f* = 1.00, *p* = 0.99, pη^2^ = 0.001) or time (*f* = 2.95, *p* = 0.08, pη^2^ = 0.45) and no interaction effect was identified (*f* = 1.69, *p =* 0.23, pη^2^ = 0.32) (Fig. 3d). In addition, NaHCO_3_ ingestion resulted in faster 40 km cycling TT performance (*f* = 4.61, *p* = 0.037, pη^2^ = 0.26) in comparison to familiarisation TTs (MD = ~ 62 s, *p* = 0.03); however, the familiarisation TT performance time did not differ from that of the placebo (MD = ~ 16 s, *p* = 0.49). Moreover, five successful detections of perceived ingested treatment were observed whereas most of the responses were comprised of unsure (*n* = 10) or incorrect (*n* = 13). There was no significant trial order effect between experimental trials (*t* = 0.12, *p* = 0.46, *g* = 0.01), nor were there any changes in recorded FiO_2_ (MD = ~ 0.10%, *t* = 1.39, *p* = 0.204, *g* = 0.14), environmental temperature (MD = ~ 0.20 °C, *t* = 0.46, *p* = 0.65, *g* = 0.11), or relative humidity (RH) (MD = ~ 0.64%, *t* = 0.56, *p* = 0.29, *g* = 0.24) between the trials.

### Blood acid–base responses

**Fig. 4:**
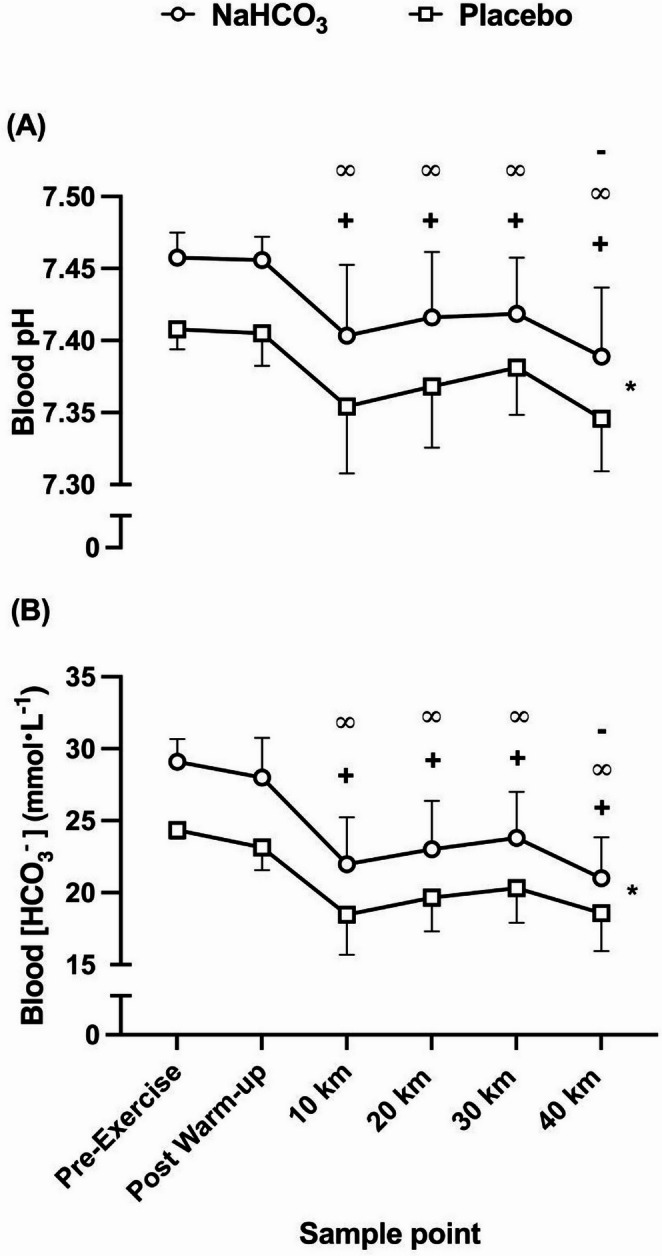
Mean (± SD) blood pH (A) and blood bicarbonate [HCO_3_^−^] (B) responses to sodium bicarbonate (NaHCO_3_) and placebo ingestion during the time trials. **+** denotes significant decrease from first timepoint (pre-exercise) (*p* < 0.01), **∞** denotes significant decrease from second timepoint (post warm-up) (*p* < 0.01), - denotes significant decrease from fifth timepoint (30 km) (*p* < 0.05), * denotes significant condition difference at all timepoints (*p* < 0.001)

The ingestion of NaHCO_3_ raised blood HCO_3_^−^ by 4.8 ± 1.0 mmol·L^− 1^ in comparison to placebo at pre-exercise and remained elevated throughout exercise (Fig. 4b) (*f* = 64.13, *p* = < 0.001, pη^2^ = 0.84). Additionally, blood pH was significantly increased following NaHCO_3_ ingestion (*f* = 48.47, *p* = < 0.001, pη^2^ = 0.79) in comparison to placebo at every timepoint (Fig. 4a). Both blood variables followed similar patterns throughout the course of the TT, which resulted in blood HCO_3_^−^ and blood pH decreasing from pre-exercise to the first quartile of the TT, followed by an increase from 10 km to 30 km, and a subsequent decrease from 30 to 40 km (blood HCO_3_^−^, *f* = 17.26, *p* = < 0.001, pη^2^ = 0.92; blood pH, *f* = 9.76, *p* = 0.002, pη^2^ = 0.84). This resulted in a significant condition*time interaction effect for blood HCO_3_ (*f* = 6.38, *p* = 0.02, pη^2^ = 0.35); however, there was no interaction effect observed for blood pH (*f* = 0.46, *p* = 0.27, pη^2^ = 0.46).

### Blood–gas and oximetry responses

**Fig. 5:**
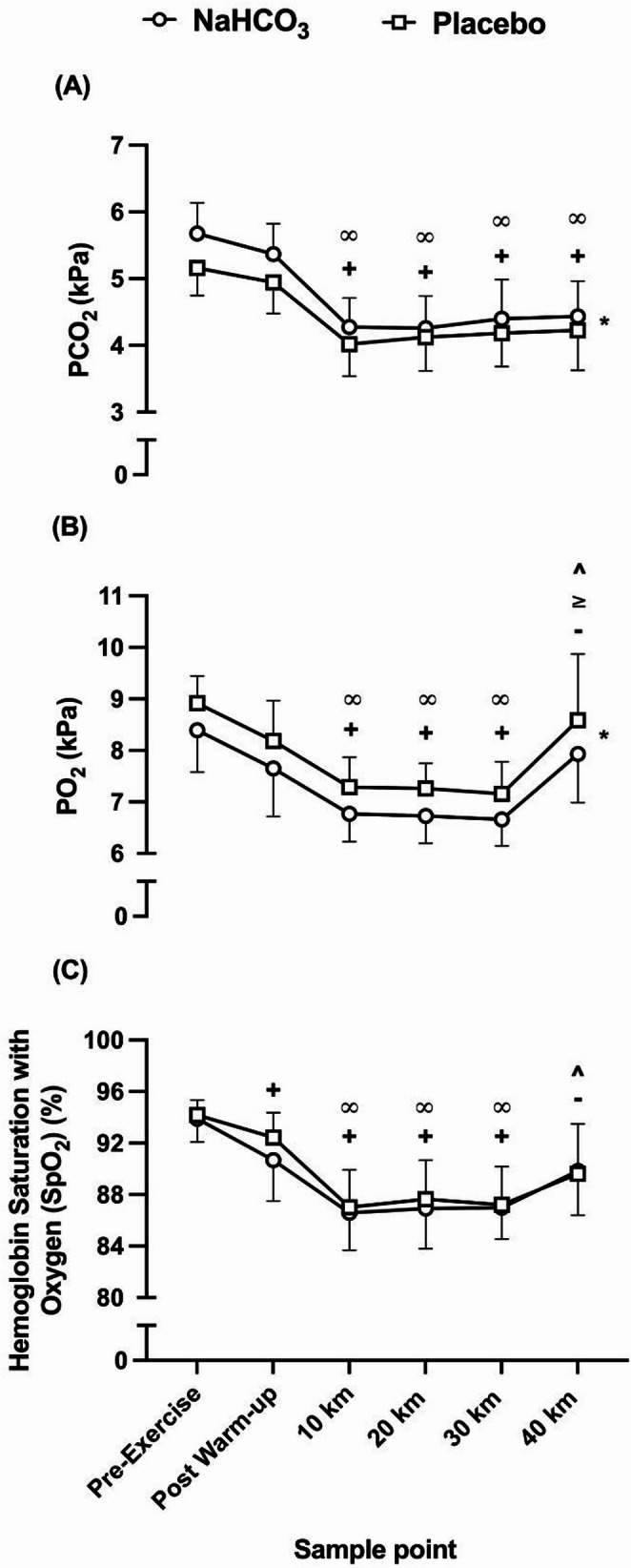
Mean (± SD) PCO_2_ (A), PO_2_ (B), and hemoglobin saturation with oxygen (SpO_2_) (C) responses to sodium bicarbonate (NaHCO_3_) and placebo ingestion during the time trials. **+** denotes significant decrease from first timepoint (pre-exercise) (*p* < 0.01), **∞** denotes significant decrease from second timepoint (post warm-up) (*p* < 0.01), **-** denotes significant increase from third timepoint (10 km) (*p* < 0.01), ≥ denotes significant increase from fourth timepoint (20 km) (*p* < 0.05), **^** denotes significant increase from fifth timepoint (30 km) (*p* < 0.01). * denotes significant difference between conditions (*p* < 0.01)

The ingestion of NaHCO_3_ increased PCO_2_ in comparison to placebo (*f* = 10.85, *p* = 0.007, pη^2^ = 0.50), whereby PO_2_ was reduced at all time points following NaHCO_3_ ingestion in contrast to placebo (*f* = 14.86, *p* = 0.005, pη^2^ = 0.65) (Fig. 5ab). Each blood-gas sample followed similar patterns from pre-exercise to the end of the TT (PCO_2_, *f* = 25.90, *p = <* 0.001 pη^2^ = 0.95; PO_2_, *f* = 0.65, *p* = 0.006, pη^2^ = 0.96); however, there were no significant interaction effects for either blood gas parameter (PCO_2_, *f* = 1.91, *p* = 0.21, pη^2^ = 0.58; PO_2_, *f* = 0.45, *p* = 0.80, pη^2^ = 0.36). Furthermore, SpO_2_ did not differ between conditions (*f =* 2.37, *p* = 0.15, pη^2^ = 0.15) (Fig. 5c), but it did change over time, decreasing from pre-exercise to 30 km, followed by an increase at the end of the TT (*f* = 29.97, *p* = < 0.001, pη^2^ = 0.94). There was no significant interaction effect observed for SpO_2_ (*f* = 2.18, *p* = 0.15, pη^2^ = 0.55).

### Blood lactate, electrolyte, and strong-ion difference responses

**Fig. 6:**
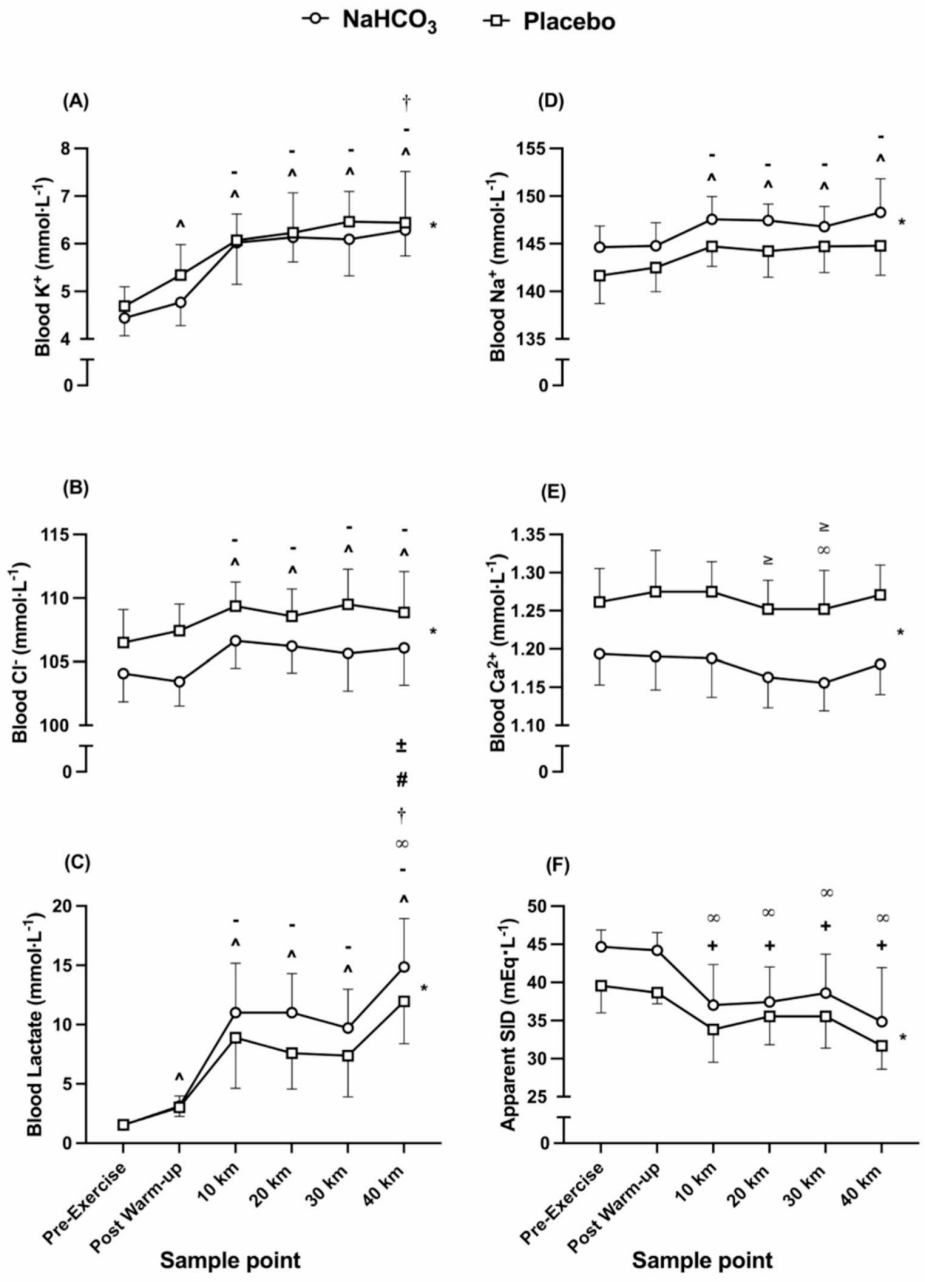
Mean (± SD) blood potassium (K^+^) (A), chloride (Cl^−^) (B), blood lactate (C), sodium (Na^+^) (D), calcium (Ca^2+^) (E), and apparent strong ion difference (SID) (F) responses during the time trials (TTs) following sodium bicarbonate (NaHCO_3_) and placebo ingestion. **^** denotes significant increase from first timepoint (pre-exercise) (*p* < 0.01), **-** denotes significant increase from second timepoint (post warm-up) (*p* < 0.05), † denotes significant increase from third timepoint (10 km) (*p* < 0.05), + denotes significant decrease from first timepoint (pre-exercise) (*p* < 0.05), **∞** denotes significant decrease from second timepoint (post warm-up) (*p* < 0.01), ≥ denotes significant decrease from third timepoint (10 km) (*p* < 0.05) **±** denotes significant increase from fifth timepoint (30 km) (*p* < 0.05), # denotes significant increase from fourth timepoint (20 km) (*p* < 0.01), * denotes significant difference between conditions (*p* < 0.01)

Pre-exercise and post warm-up blood lactate concentration did not differ between TTs (Fig. 6c), but ingestion of NaHCO_3_ resulted in an increase in blood lactate concentration across all timepoints throughout the 40 km TT in comparison to placebo (*f* = 16.34, *p* = 0.001, pη^2^ = 0.56), resulting in several interaction effects (*f* = 7.03, *p* = < 0.001, pη^2^ = 0.35). Despite this condition difference, blood lactate responses followed a similar pattern across both TTs, increasing from pre-exercise to post warm-up, remaining unchanged from 10 to 30 km, followed by a rise at the end of the TT (*f* = 62.30, *p* = < 0.001, pη^2^ = 0.97). The ingestion of NaHCO_3_ increased blood Na^+^ concentration (Fig. 6d) in comparison to placebo (*f* = 23.19, *p* = < 0.001, pη^2^ = 0.64), and followed a similar pattern in both trials (*f* = 26.48, *p* = < 0.001, pη^2^ = 0.94), increasing from pre-exercise and post warm-up whilst remaining unchanged throughout each 40 km TT. Contrastingly, the blood Ca^2+^ concentration (Fig. 6e) was reduced following NaHCO_3_ ingestion at all timepoints (*f* = 223.56, *p* = < 0.001, pη^2^ = 0.95); however, the pattern of blood Ca^2+^ was similar across both TTs (*f* = 3.91, *p* = 0.037, pη^2^ = 0.69), reducing its concentration from 10 to 30 km and remained similar throughout the rest of the TT. Furthermore, blood K^+^ concentration (Fig. 6a) was reduced at all timepoints following NaHCO_3_ ingestion (*f* = 5.07, *p* = 0.042, pη^2^ = 0.28), and increased significantly from pre-exercise to the end of the TT in both trials (*f* = 58.33, *p* = < 0.001, pη^2^ = 0.97). This was similar to blood Cl^−^ (Fig. 6b), whereby its concentration was reduced following NaHCO_3_ ingestion (*f* = 24.66, *p* = < 0.001, pη^2^ = 0.66) and significantly increased from pre-exercise to 10 km and remained similar for the rest of the TT in both trials (*f* = 14.32, *p* = < 0.001, pη^2^ = 0.89). There was no interaction effect for either of these electrolytes (Na^+^, *f* = 1.02, *p* = 0.46, pη^2^ = 0.46; Cl^−^, *f* = 2.64, *p* = 0.098, pη^2^ = 0.59; Ca^2+^, *f* = 0.81, *p* = 0.57, pη^2^ = 0.31; K^+^, *f* = 0.78, *p* = 0.59, pη^2^ = 0.30). The resultant shifts in these ions ultimately led to a greater apparent SID (Fig. 6f) following NaHCO_3_ ingestion (*f* = 22.02, *p* = < 0.001, pη^2^ = 0.63), which followed a similar pattern in both TTs (*f* = 9.16, *p* = 0.002, pη^2^ = 0.84), reducing from pre-exercise and post warm-up to the end of the TT. There were no interaction effects at any timepoint for apparent SID (*f* = 2.58, *p* = 0.103, pη^2^ = 0.59).

### Cardiorespiratory and perceptual responses

**Fig. 7:**
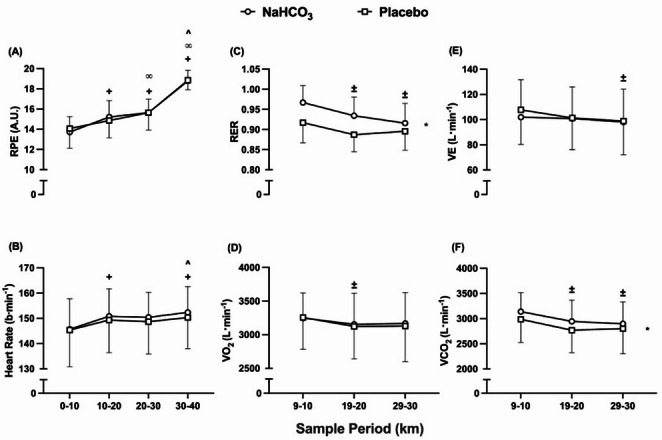
Mean (± SD) (A) Rating of Perceived Exertion (RPE) (A), heart rate (B), respiratory exchange ratio (RER) (B), VO_2_ (D), VE (E), and VCO_2_ (F) responses during the time trials following sodium bicarbonate (NaHCO_3_) and placebo ingestion. + denotes significant increase from first timepoint (0–10 km) (*p* < 0.05), **∞** denotes significant increase from second timepoint (10–20 km) (*p* < 0.01), **^** denotes significant increase from third timepoint (20–30 km) (*p* < 0.05), **±** denotes significant decrease from first timepoint (9–10 km) (*p* < 0.05), * denotes significant difference between conditions (*p* < 0.05)

The VO_2_, heart rate, and RPE responses (Fig. 7 abd) were unaffected by NaHCO_3_ ingestion throughout the TT (VO_2_, *f* = 0.179, *p* = 0.68, pη^2^ = 0.014; heart rate, *f* = 0.41, *p* = 0.53, pη^2^ = 0.03; RPE, *f* = 0.10, *p* = 0.92, pη^2^ = 0.001). Despite this, VCO_2_ (Fig. 7c) was elevated after pre-exercise alkalosis (*f* = 16.81, *p* = 0.001, pη^2^ = 0.56), and there was also a consequent elevation of RER (Fig. 7f) (*f* = 7.40, *p* = 0.018, pη^2^ = 0.36). Minute ventilation (Fig. 7e) was unaffected by condition (*f* = 0.94, *p* = 0.35, pη^2^ = 0.067), but followed a similar pattern across the TTs, decreasing from 9 to 10 km to 29–30 km (*f* = 4.87, *p* = 0.028, pη^2^ = 0.45). Heart rate followed a familiar pattern across both TTs (*f* = 26.47, *p* = < 0.001, pη^2^ = 0.88), increasing from the 10–20 km, followed by an additional increase from 30 to 40 km. Additionally, RPE changed over time throughout both TTs (*f* = 78.98, *p* = < 0.001, pη^2^ = 0.96), increasing at each quartile from 10 to 40 km. Furthermore, VCO_2_ (*f* = 15.70, *p* = < 0.001, pη^2^ = 0.72) and RER (*f* = 15.02, *p* = < 0.001, pη^2^ = 0.72) responded in a comparable manner throughout each TT, with each response decreasing from 9 to 10 km to 19–20 km to 29–30 km. This was similar to VO_2_, whereby these responses changed over time throughout both TTs (*f* = 4.68, *p* = 0.031, pη^2^ = 0.44), decreasing from 9 to 10 km to 19–20 km. There were no interaction effects for either cardiorespiratory or perceptual response (VO_2_, *f* = 0.41, *p* = 0.67, pη^2^ = 0.44; heart rate, *f =* 0.60, *p* = 0.63, pη^2^ = 0.14; VCO_2_, *f* = 1.48, *p* = 0.27, pη^2^ = 0.20; RER, *f* = 2.15, *p* = 0.16, pη^2^ = 0.26; VE, *f* = 0.87, *p* = 0.94, pη^2^ = 0.13).

### Gastrointestinal responses

**Fig. 8:**
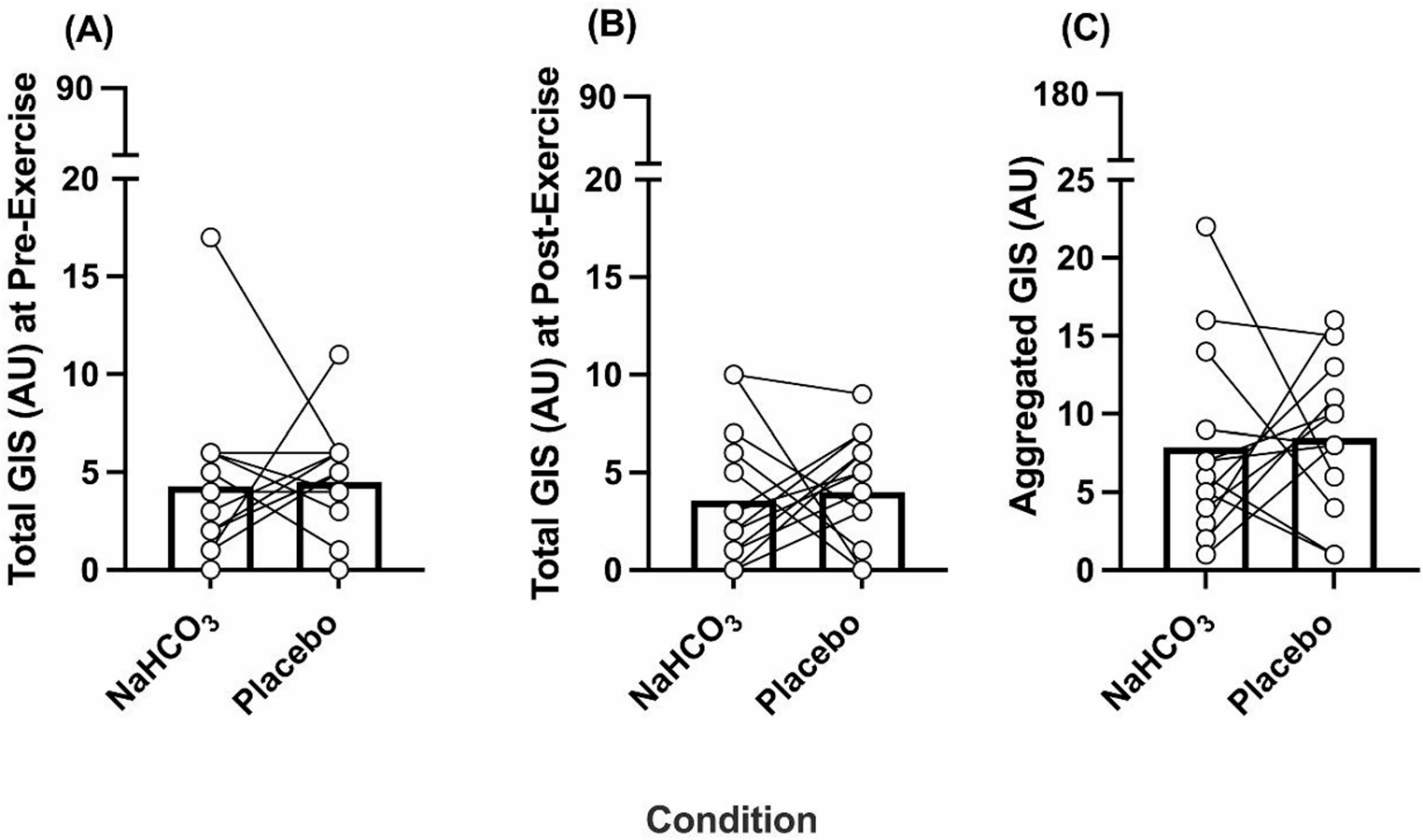
Individual and mean gastrointestinal symptoms (GIS) (AU) responses following the ingestion of sodium bicarbonate (NaHCO_3_) and placebo at pre-exercise (A), post-exercise (B), and aggregated GIS (C)

After ingestion and prior to exercise, there were no differences in GIS between conditions (NaHCO_3_, 60 AU, Placebo, 63 AU; *z* = 0.23, *p* = 0.82, *r =* 0.06) (Fig. 8a). This was also the case at the end of both TTs, whereby the total GIS did not differ between NaHCO_3_ and placebo (NaHCO_3_, 50 AU, Placebo, 56 AU; *z* = 0.42, *p* = 0.67, *r* = 0.11) (Fig. 8b). Furthermore, there were no significant differences in peak severity score at pre-exercise (*z =* 1.04, *p* = 0.31, *r* = 0.26) or post exercise (*z* = 0.02, *p* = 0.92, *r* = 0.02) (Table 1). In addition to this, total aggregated GIS was not elevated by NaHCO_3_ ingestion, compared to the placebo (NaHCO_3_ 110 AU, Placebo, 119 AU; *z* = 0.38, *p* = 0.71, *r* = 0.10) (Fig. 8c).

**Table 1:** The most severe individual GI symptom score reported at each timepoint (pre-exercise, post-exercise). Symptom scores are displayed in parenthesis and are expressed as arbitrary units (AU) from 0 representing “no symptom” and 10 representing “most severe symptom”

	NaHCO_3_		Placebo	
Participant	Pre-exercise	Post-exercise	Pre-exercise	Post-exercise
1	Stomach-bloating (6)	Stomach-ache, stomach bloating (2)	Stomach-ache, stomach cramp (3)	Nil (0)
2	Flatulence (2)	Nil (0)	Flatulence (1)	Nil (0)
3	Nil (0)	Belching (3)	Nil (0)	Belching (1)
4	Diarrhoea (2)	Stomach-cramp (3)	Stomach bloating (2)	Nil (0)
5	Stomach-ache (2)	Nausea (4)	Belching, stomach-ache, nausea (1)	Nausea, flatulence (2)
6	Flatulence (4)	Stomach cramp, bloating, belching (1)	Belching (2)	Belching (2)
7	Flatulence (1)	Stomach cramp (1)	Stomach bloating (1)	Flatulence (2)
8	Belching (1)	Stomach-ache, flatulence (1)	Stomach-cramp (3)	flatulence (3)
9	Stomach-cramp (2)	Stomach-ache, flatulence (1)	Stomach-cramp (1)	Stomach bloating (3)
10	Stomach-cramp, stomach bloating (1)	Flatulence (3)	Belching (1)	Stomach cramping (2)
11	Bowel urgency (2)	Stomach-ache (1)	Bowel urgency (1)	Flatulence, stomach-ache (1)
12	Flatulence, stomach-cramp (1)	Nil (0)	Stomach-ache (1)	Stomach-ache (3)
13	Stomach-ache (1)	Nil (0)	Stomach-bloating (1)	Nausea (1)
14	Nausea (1)	Nausea (1)	Flatulence (1)	Flatulence (1)

## Discussion

The present study aimed to determine the effect of 0.3 g·kg^− 1^ BM NaHCO_3_ mini-tablets ingested in a CHO hydrogel on 40 km cycling TT performance in an ~ 1850 m simulated altitude environment. This is the first study to establish that ingesting NaHCO_3_ in the form of mini-tablets in a CHO hydrogel 90 min prior to exercise can enhance 40 km cycling TT performance in acute hypoxia, demonstrated by a 1.2% improvement with minimal GIS. This performance improvement was likely a result of an improved extracellular buffering capacity, enhanced gross efficiency, and/or an improved apparent SID. This enhancement in 40 km cycling TT performance in acute hypoxia represents a small but meaningful change in performance.

In addition to the observed ergogenic benefits following NaHCO_3_ ingestion, blood HCO_3_^−^ was elevated at each timepoint, including a 4.8 ± 1.0 mmol·L^− 1^ raise at pre-exercise when compared to the placebo trial. This increase in blood HCO_3_^−^ is comparable to previous research investigating enteric-coated NaHCO_3_ on prolonged high-intensity exercise performance as demonstrated by Leach et al. (2023), where these authors observed a 3.7 mmol·L^− 1^ increase prior to enhancing 16.1 km cycling TT performance by 2.5%. Interestingly, these increases are correspondingly lower than the frequently suggested 5 mmol·L^− 1^ metabolic threshold most likely to generate a performance enhancing effect (Carr et al. 2011). Contrary to the current investigation, other studies investigating the effect of NaHCO_3_ mini-tablets in a CHO hydrogel on exercise performance have reported greater pre-exercise alkalotic increases such as 7.1 mmol·L^− 1^ (Gough and Sparks 2024b) and 5.7 mmol·L^− 1^ (Shannon et al. 2024) whilst demonstrating improvements in 4 km and 40 km cycling TT performance, respectively. However, these earlier studies individualised the timing of 0.3 g·kg^− 1^ BM NaHCO_3_ ingestion to ensure that exercise commenced at peak blood alkalosis, thereby accounting for the inter-individual variability in time to reach peak blood HCO_3_^−^ (Gough et al. 2017) which may explain the higher pre-exercise blood alkalosis concentrations when compared to the current investigation. In contrast, the present study implemented a 90 min ingestion strategy, carefully chosen based on consistent peak blood HCO_3_^−^ responses observed by Gough and Sparks (2024b), denoting that each participant (*n* = 10) reached a 5 mmol·L^− 1^ increase in blood HCO_3_^−^ by 90 min, which is also a timepoint that aligns with the manufacturer’s recommended ingestion guidelines. Whilst individualising NaHCO_3_ ingestion may optimise pre-exercise blood alkalosis, the current investigation’s implementation of a standardised 90 min ingestion strategy using NaHCO_3_ mini tablets offers a more simplified and externally valid consumption strategy, subsequently enabling greater feasibility for athletes and practitioners to incorporate into important competition preparation.

Although a performance enhancement was observed following NaHCO_3_ ingestion, as demonstrated by a greater power output, speed, and shorter time to finish, interestingly, cadence, RPE, heart rate, and VO_2_ remained unchanged, suggesting enhanced torque, gross efficiency, and contractility. This is comparable to recent work investigating NaHCO_3_ ingestion on high-intensity exercise (Shannon et al. 2024), but contrasts with others that have used traditional NaHCO_3_ ingestion forms (Voskamp et al. 2020). It is possible that the enhancement in gross efficiency and contractility was aided by an interaction between the reported peripheral and central acting mechanisms of NaHCO_3_ ingestion (Siegler and Marshall 2015; Siegler et al. 2016). Previous work has demonstrated improvements in the rate of torque development and increased voluntary activation (gross estimate of central drive) during a 2 min multiple voluntary contraction (MVC) (Siegler and Marshall 2015), which has been suggested to be at least, in part, due to a reduced attenuation of group III and IV afferent firing under decreased acidic conditions (Pollak et al. 2014). A reduction in these afferents because of induced alkalosis could therefore minimise the negative feedback from the muscle whilst maintaining drive to the motor neurons (Swank and Robertson 2002) and subsequently diminish the localised pain in the fatiguing muscle (Robertson et al. 1992). Nonetheless, because the current investigation did not incorporate an estimated measure of central drive, it is not possible to elucidate whether the interplay between the central and peripheral mediated responses following NaHCO_3_ ingestion during a 2 min MVC can explain the present study’s results in prolonged high-intensity exercise. Clearly, further research is necessary to ascertain if performance enhancements in prolonged high-intensity exercise following NaHCO_3_ ingestion without incurring a change in cadence, RPE, heart rate or VO_2_ is supported by reducing a combination of central and peripheral components of exercise-induced fatigue.

Throughout the 40 km cycling TT, NaHCO_3_ ingestion raised VCO_2_ and RER in comparison to placebo. These elevated respiratory responses throughout the 40 km cycling TT following NaHCO_3_ ingestion are likely a result of an elevated extracellular blood buffering capacity, and as a result, leads to an increased release of CO_2_ through carbonic acid, similar to earlier research (Stephens et al. 2002; Cox and Jenkins 1994). Additionally, blood lactate was elevated throughout TT following NaHCO_3_ ingestion, likely a result of enhanced glycolytic flux (Hollidge-Horvat et al. 2000) combined with the enhanced power output observed in the present study. Heightened blood lactate concentrations following induced alkalosis reflects greater lactate and proton efflux from the muscle to the extracellular space serving as an indirect marker of improved glycolytic rate during the TT (Messonnier et al. 2007; McNaughton et al. 2008). The ingestion of NaHCO_3_ widens the pH gradient between the extracellular and intracellular compartments, which enables a faster release of lactate and H^+^ ions from the intracellular space to the blood via the lactate/H^+^ co-transporters (Requena et al. 2005). The ingestion of NaHCO_3_ also shifted the movement of each electrolyte and elevated the apparent SID from pre-exercise to the end of the TT. Muscle fatigue, as a result of high-intensity exercise, can be attributed to the disturbances in SID (Sostaric et al. 2006) by diminishing Na^+^, K^+^-ATPase activity, which thereby weakens the cell-membrane excitability (Fraser et al. 2002). As such, extracellular Cl^−^ and K^+^ were reduced following NaHCO_3_ ingestion and throughout exercise, comparable to previous research (Shannon et al. 2024; Gough et al. 2018a), which may be resultant of a greater uptake of these electrolytes in the muscle that may protect excitation-contraction coupling (Sostaric et al. 2006). Since the current study only measured these ionic changes in the extracellular space, the movement of K^+^ from the intracellular compartment and into the blood throughout prolonged high-intensity exercise in hypoxia can therefore only be speculated and provides an additional avenue for future research.

Contributing to the ergolytic effect of altitude on exercise performance when compared to exercise at sea level is likely a result of the reduced O_2_ saturation since this corresponding reduction impairs the O_2_ delivery to the active musculature, subsequently diminishing the overall aerobic capacity (Bassett and Howley 2000). Indeed, Chapman et al. (2011) demonstrated that the preservation of arterial oxyhemoglobin saturation (SaO_2_) improves 3000 m running performance in moderate hypoxia (~ 3000 m). Comparably, earlier research observed enhancements in 2000 m rowing performance following infused intravenous NaHCO_3_ through the radial artery whilst also improving SaO_2_ when compared to placebo (Nielsen et al. 2002); however, NaHCO_3_ ingestion had no influence on blood SpO_2_ during the current investigation. Nonetheless, the placebo adopted by Nielsen et al. (2002) was an infused equimolar dose of saline which is known to cause metabolic acidosis (Kellum 2002) and could therefore reduce the O_2_ delivery and indirectly attenuate SaO_2_, creating uncertainty if NaHCO_3_ can protect markers of O_2_ saturation during exercise. Clearly, further research is necessary to establish the effect of NaHCO_3_ compared to a variety of placebos on O_2_ saturation during high-intensity exercise performance.

The present study utilised a form of NaHCO_3_ that aims to reduce GIS in comparison to traditional ingestion forms of the extracellular buffer (aqueous solution, capsules). As such, the current investigation demonstrated no significant differences in GIS (aggregated, total, peak) in comparison to a maltodextrin and appearance-matched placebo, which is similar to recent work analysing this form of NaHCO_3_ on exercise performance (Gough and Sparks 2024b; Shannon et al. 2024). The proposed mechanism by which this form of NaHCO_3_ achieves a reduction in GIS compared to traditional ingestion forms is likely a result of a reduced interaction between the NaHCO_3_ mini-tablets and stomach acid since these mini-tablets are suggested to pass through the pyloric sphincter (Gough and Sparks 2024a). This contrasts with long-established methods of ingesting NaHCO_3_, such as aqueous solution and gelatin capsules, whereby the interaction with stomach acid is greater, which subsequently heightens the GIS response. Given the magnitude of GIS associated with traditional NaHCO_3_ forms (Hilton et al. 2020b), which is suggested to impair performance (Cameron et al. 2010; Kahle et al. 2013; Saunders et al. 2014b) and prevent athletes and practitioners from using this agent altogether, NaHCO_3_ mini-tablets in a CHO hydrogel may offer a more practical alternative considering its minimal GIS response. Interestingly, some GIS was also present in the placebo trial, despite replacing the NaHCO_3_ mini-tablets with maltodextrin mini-tablets. The reason for some GIS during the placebo trial remains unclear; however, it may have resulted from participants not having previously trained with the CHO hydrogel after their pre-exercise meal (albeit replicated before each visit), which could exacerbate the GIS response whilst cycling due to the position on the ergometer.

### Future directions and limitations

The present study had some limitations that generally concern the participants that have been recruited. This means that the present findings are only are only generalisable to a trained male cyclist population. Therefore, it is not possible to determine whether these results are transferable to trained female cyclist population, since sex-differences in extracellular buffering capacity have been observed following NaHCO_3_ ingestion (Durkalec-Michalski et al. 2025). Future research should therefore consider examining the effect of NaHCO_3_ ingestion on prolonged high-intensity exercise performance in acute normobaric hypoxia in trained female cyclists. An additional limitation of the current study is the exclusion of elite cyclists. Recruiting and testing elite cyclists presents a substantial and logistical challenge for researchers (i.e. scheduling around the training and competition commitments), and therefore, the current investigation focused exclusively on trained male cyclists. Consequently, it is unclear whether the observed 1.2% improvement in 40 km cycling TT performance in acute normobaric hypoxia would result in similar performance improvements in elite male cyclists, a finding that could be particularly meaningful given the importance of marginal gains in elite cycling. Future research should aim to extend these findings within an elite cyclist sample population to elucidate whether similar ergogenic effects can be observed.

## Conclusion

The principal findings of this study are that the ingestion of NaHCO_3_ mini-tablets in a CHO hydrogel improves 40 km cycling TT performance in an ~ 1850 m simulated altitude environment with minimal difference in GIS when compared to placebo. It is likely that the observed improvement in 40 km cycling TT performance following NaHCO_3_ ingestion is a result of an improved extracellular buffering capacity, an increase in SID throughout the TT, and/or a lower relative oxygen cost which suggests enhanced gross efficiency. Athletes, practitioners, and nutritionists should therefore consider the use of NaHCO_3_ ingestion at altitude prior to prolonged high-intensity exercise to minimise the deleterious physiological effects in this environment.

## Data Availability

All data is available upon request to the corresponding author.

## Abbreviations

AU: Arbitrary unit
Ca^2+^: Blood calcium ion
Cl^−^: Blood chloride ion
CHO: Carbohydrate
FiO_2_: Fraction of inspired oxygen
GIS: Gastrointestinal symptoms
H^+^: Hydrogen cation
HCO_3_^−^: Bicarbonate anion
K^+^: Blood potassium ion
Na^+^: Blood sodium ion
NaHCO_3_: Sodium bicarbonate
PCO_2_: Partial pressure of carbon dioxide
PO_2_: Partial pressure of oxygen
RPE: Rating of perceived exertion
SaO_2_: Arterial oxygen saturation
SID: Strong ion difference
SpO_2_: Hemoglobin with oxygen saturation
TT: Time trial
VO_2Peak_: Peak oxygen uptake
